# Displacement Study of a Large-Scale Freeform Timber Plate Structure Using a Total Station and a Terrestrial Laser Scanner

**DOI:** 10.3390/s20020413

**Published:** 2020-01-11

**Authors:** Anh Chi Nguyen, Yves Weinand

**Affiliations:** Laboratory for Timber Constructions (IBOIS), École Polytechnique Fédérale de Lausanne, GC H2 711, Station 18, CH-1015 Lausanne, Switzerland

**Keywords:** timber plate structures, total station, terrestrial laser scanning, point cloud registration, finite element spring model

## Abstract

Recent advances in timber construction have led to the realization of complex timber plate structures assembled with wood-wood connections. Although advanced numerical modelling tools have been developed to perform their structural analysis, limited experimental tests have been carried out on large-scale structures. However, experimental investigations remain necessary to better understand their mechanical behaviour and assess the numerical models developed. In this paper, static loading tests performed on timber plate shells of about 25 m span are reported. Displacements were measured at 16 target positions on the structure using a total station and on its entire bottom surface using a terrestrial laser scanner. Both methods were compared to each other and to a finite element model in which the semi-rigidity of the connections was represented by springs. Total station measurements provided more consistent results than point clouds, which nonetheless allowed the visualization of displacement fields. Results predicted by the model were found to be in good agreement with the measurements compared to a rigid model. The semi-rigid behaviour of the connections was therefore proven to be crucial to precisely predict the behaviour of the structure. Furthermore, large variations were observed between as-built and designed geometries due to the accumulation of fabrication and construction tolerances.

## 1. Introduction

Over the past decades, geometrically complex and large-span timber structures have been achieved thanks to advances in both engineering and architecture. Significant research studies have especially focused on the development of timber plate structures using wood-wood connections inspired by traditional carpentry joints. These structures consist of a large number of singular planar elements connected at their edges by joints fully integrated in the plates. Research prototypes as well as building-scale assemblies have been built using computer-aided design (CAD) programming and digital fabrication tools [1,2,3,4,5,6]. Various numerical models, mostly based on the finite element method, have been developed for their structural analysis but only limited experimental tests have been carried out on large-scale structures. However, experimental investigations remain necessary to better understand their mechanical behaviour and assess the numerical models developed. Furthermore, full-scale tests are required when physical models cannot be reduced to a small portion of the structure to analyse its global behaviour or when small-scale models cannot duplicate the behaviour of the real-size structure [7,8]. Such limitations can result from material variability and execution tolerances, which might have a high influence on the mechanical behaviour of the global structure [7]. This is particularly the case for assemblies with a large number of complex joints, contributing to significant uncertainties [7,9].

Full-scale experimental tests can be carried out either in the laboratory or in the field. Since timber plate structures using wood-wood connections have been developed relatively recently, only few building-scale structures have been built and laboratory tests have thus mostly been carried out for smaller spans. For shell structures in particular, loading tests remain difficult to execute as they are designed to transmit surfacic loads such as self-weight, snow and wind loads to the supports. Several methods can be used to simulate distributed loads on them. Traditional methods consist in pressure loading, vacuum loading or discrete loading systems, usually closely-spaced [10]. Pressure loading can be achieved through gravity using water or sand with a possible additional surcharge load. It can also be obtained with a gas pressure system or with a sealed air bag. Similarly to air pressure loading, vacuum loading is restricted to structures with continuously supported and sealed edges. Discrete loading can be carried out using suspended dead weights or through the use of a whiffle-tree system. More recently, innovative pulley-based loading systems have also been developed [9,11]. Their advantages are their high flexibility, safety of the method and absence of variation of the applied load with deformation of the structure. Onsite, however, not all laboratory test methods can be applied and experimental tests on large-scale shells have not been reported in the literature. Examples of static loading tests achieved through pressure and discrete loading systems can nonetheless be found for timber frame and gridshell structures respectively. In Doudak et al. [12], tests were executed on a 8.5 × 17 m single-storey timber frame structure by applying a load of 3.3 kN/m${}^{2}$ on patches of the roof using dead weights consisting of shingle bundles and by measuring the reaction forces. For the 60 × 60 m span *Multihalle* timber gridshell in Mannheim, Germany designed by Frei Otto, a discrete loading system, consisting of garbage bins hanging on the gridshell and filled with water, has been used. In this study, tests were performed with the objective of assessing the accuracy of the calculations by comparing the deflections measured at 13 points on the structure with the corresponding computed deflections [13].

Instrumentation for experimental testing depends on the variables of interest. In particular, displacements provide key information to better understand the behaviour of loaded structures as they give an indication on the stiffness of the structural system. Displacements measurements can be acquired using numerous techniques, such as global positioning system (GPS), linear variable differential transformers (LVDT), laser Doppler vibrometer (LDV) and total stations, which are examples of conventional equipment [14]. Each of them is characterized by different data acquisition methods and accuracy, such that they are not suitable for all applications. GPS sensors can measure both static and dynamic displacements but do not provide sufficient accuracy for displacements under 1 cm [15]. LVDTs are both accurate and low cost; however, as they measure contacting displacements between a limited number of target points on the structure and fixed reference points, they are unpractical for large-scale structures and usually require the presence of additional temporary structures [16]. LDVs have the advantage to measure non-contact displacements with comparable accuracy but are costly devices requiring high incident angles between the laser beams and the vibrating surface [16,17]. Total stations have been used to measure displacements in numerous case studies because of their ease of use and accuracy in the millimetre range [18,19,20]. However, they allow measurements on a limited number of target points. More recently, three-dimensional (3D) terrestrial laser scanning has also been used for several applications [21,22,23,24]. With this technique, coordinates of millions of points forming a point cloud are collected. Massive sampling data in the few millimetres range can be obtained from laser scans but their processing is more complicated.

This paper focuses on a recent case study, namely the Annen Plus SA head office in Manternach, Luxembourg, composed of large-span double-layered double-curved timber plate shells recently developed [25,26]. Although a numerical model has been proposed and automated for both small- and large-scale structures, it has been validated against experimental investigations on small-scale prototypes only [26]. Therefore, the aim of the present study is to assess the numerical model developed through experimental investigations on large-scale structures and evaluate possible methods to acquire displacements of the loaded structure, using a total station and a terrestrial laser scanner. The design of the structure and loading procedure are first described in Section 2, followed by the displacement measurement methods and numerical modelling in Section 3 and Section 4 respectively. Both methods are then compared to each other and to the numerical model in Section 5.

## 2. Experimental Tests

### 2.1. Structural Design

**Figure 1:**
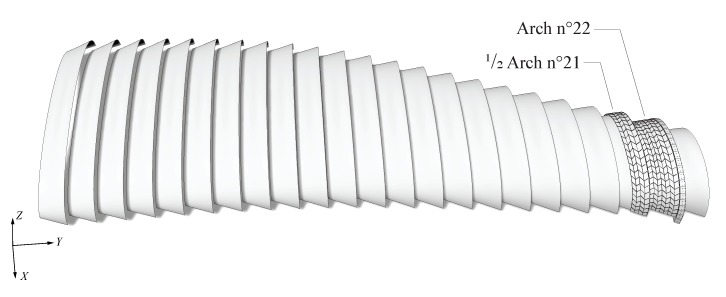
Illustration of the Annen Plus SA head office project in Manternach, Luxembourg consisting of 23 double-layered double-curved timber plate shells. Experimental tests were carried out on prototypes of arch n° 22 and half of n° 21.

**Figure 2:**
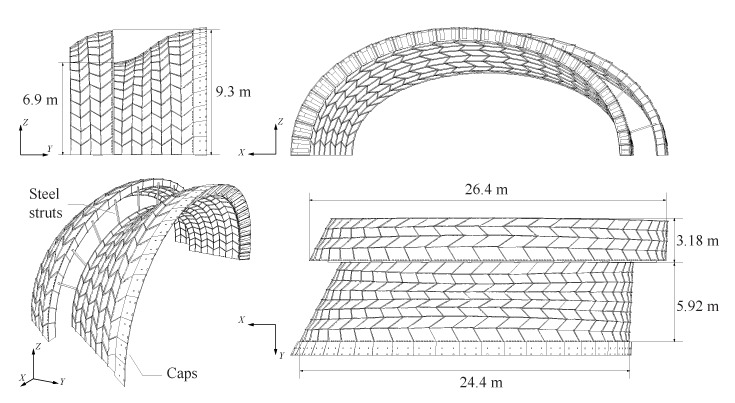
Geometry of the tested prototypes of arch n° 22 and half of n° 21.

The structure tested consisted in prototypes built for the Annen Plus SA head office project in Manternach, Luxembourg, which will accommodate 5800 m${}^{2}$ factory space and offices [25]. Its roof structure consists of 23 double-layered double-curved timber plate shells with constant height and width of 9 and 6 m respectively and spans ranging from 23 to 54 m. The second to last arch and half of its larger neighbouring arch (arch n° 22 and half of arch n° 21 respectively, as shown in Figure 1) were constructed by Annen Plus SA. Their goal was to verify the realization of the supports of the structure for the project, as well as the connection between the arches. Therefore, only half of arch n° 21 was constructed. For project purposes, a row of timber caps was added on arch n° 22 along its entire open side and the two arches were connected by ten 100 × 100 × 5 mm steel struts. The geometry of the tested prototypes, with a span of 25 m in average, is presented in Figure 2.

### 2.2. Fabrication and Assembly

The double-layered double-curved shells use an assembly system developed by Robeller et al. [25] and described in Appendix A. The structures were built from 40 mm thick Pollmeier BauBuche Q panels, which are beech laminated veneer lumber (LVL) panels, obtained by gluing fourteen layers of 3 mm thick beech peeled veneers longitudinally and cross-wised [27]. The composition of the panel is ∣∣∣−∣∣∣∣∣∣−∣∣∣ (∣ for longitudinal, − for crosswise veneer layer). The tested prototypes were made of 792 singular shaped plates forming 200 boxes for the full arch and 396 plates forming 100 boxes for the half arch. The arches with all their individual plates and different joint geometries were designed using custom developed CAD plugins. All plates of the structure were digitally fabricated by Annen Plus SA with a 5-axis computer numerical controlled (CNC) machine, using a 20 mm diameter tool for tilt angles inferior to 45° and of 25 mm for larger angles [26]. Annen Plus SA manually assembled the arches lying on their side (XZ-plane in Figure 2), lifted them up individually in one single assembly and fixed them to the supports using a crane.

### 2.3. Supports

**Figure 3:**
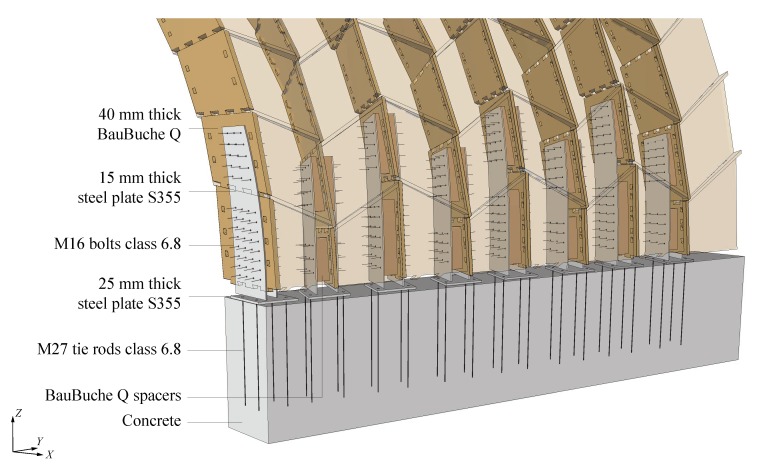
Supports of arch n° 22.

The supports of the structure are illustrated in Figure 3 for arch n° 22. On both ends of the arches, 15 mm thick steel plates were fixed to vertical plates of two first rows of boxes by M16 bolts through BauBuche Q spacers. These plates were welded to 25 mm thick steel plates, themselves fixed on concrete blocks with M27 tie rods anchored using grouting mortar.

### 2.4. Loading Procedure

**Figure 4:**
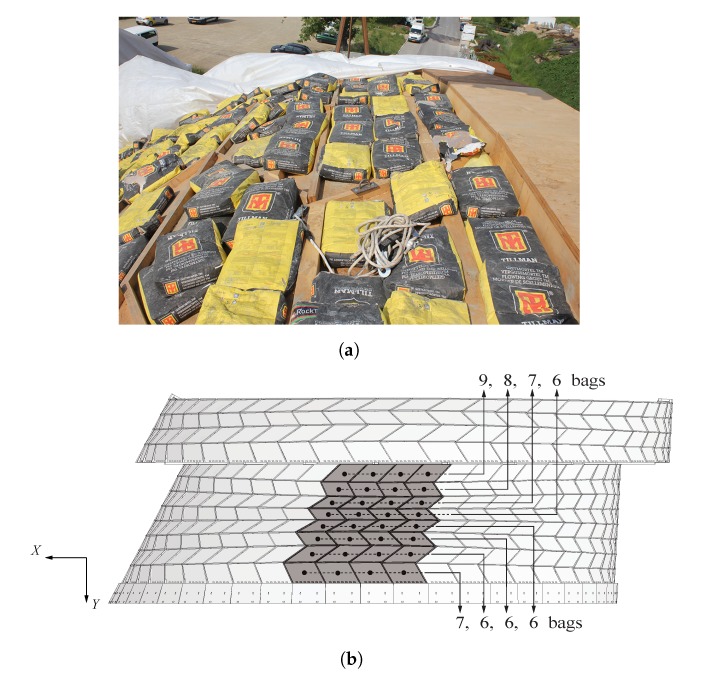
(**a**) Loading of the structure using 25 kg cement bags; (**b**) A distributing loading of 1.5 kN/m^2^ in average was applied on the zone of the structure with a lower curvature (grey area). The varying number of cement bags leading to the average load is indicated.

Static loading tests were conducted using pressure loading. Vacuum loading methods would not have been feasible because of the presence of gaps in the structure and the difficulty to seal all of its edges. Moreover, limited atmospheric pressure restricts the load magnitude that can be applied on it. Discrete point loading was discarded to avoid wind effects on the hanging masses. As illustrated in Figure 4a, pressure loading was achieved through gravity only by applying a surcharge load on top of the structure using 25 kg cement bags distributed over the top surface. The disadvantage of this method was that a large number of bags was necessary to obtain a sufficient load magnitude and the loading procedure was thus labor intensive. The top layer of the structure was only partially loaded as it corresponds to the worst load case with respect to the vertical displacements at midspan. Furthermore, additional components were not necessary to retain the bags on the structure thanks to the low curvature of the structure in the *XZ*-plane in this area. Since the objective of the test was to compare onsite measurements with the numerical model, the loading method was considered acceptable as long as the specific surcharge load was accurately implemented in the numerical model. Furthermore, loading from the top allowed to have a clear sight under the structure for displacement measurements. A non-destructive elastic test was performed by applying an average load of 1.5 kN/m${}^{2}$ on the area of the structure. The loaded area is highlighted in Figure 4b, with the indication of the number of bags placed on each box. In fact, this number was adapted depending on the loaded surface of each box to correspond to an equivalent load of approximately 1.5 kN/m${}^{2}$.

Steel struts illustrated in Figure 2 and connecting the two arches were not directly mounted. The two tests were thus performed following the same loading procedure and with the identical surcharge load applied on arch n° 22 only (as shown in Figure 4b):

- Test n° 1: arch n° 22 not connected to half of arch n° 21, which was therefore not considered in the test measurements, on 22 May 2018 and;
- Test n° 2: arch n° 22 fixed to half of arch n° 21 by steel struts, as illustrated in Figure 2, on 23 May 2018.

## 3. Instrumentation

Displacements were measured using a total station and a terrestrial laser scanner. Two sets of measurements were taken for each test, before and after loading respectively, such that four different load cases can be defined:

- Load case n° 1: test n° 1 before loading
- Load case n° 2: test n° 1 under final loading
- Load case n° 3: test n° 2 before loading
- Load case n° 4: test n° 2 under final loading

Considering the low load levels applied, the mechanical behaviour of the structure was assumed to be linear elastic, such that the effects of the load release after Load case n° 2 were discarded and no influence on the displacements measured during test n° 2 was taken into account.

### 3.1. Total Station

**Figure 5:**
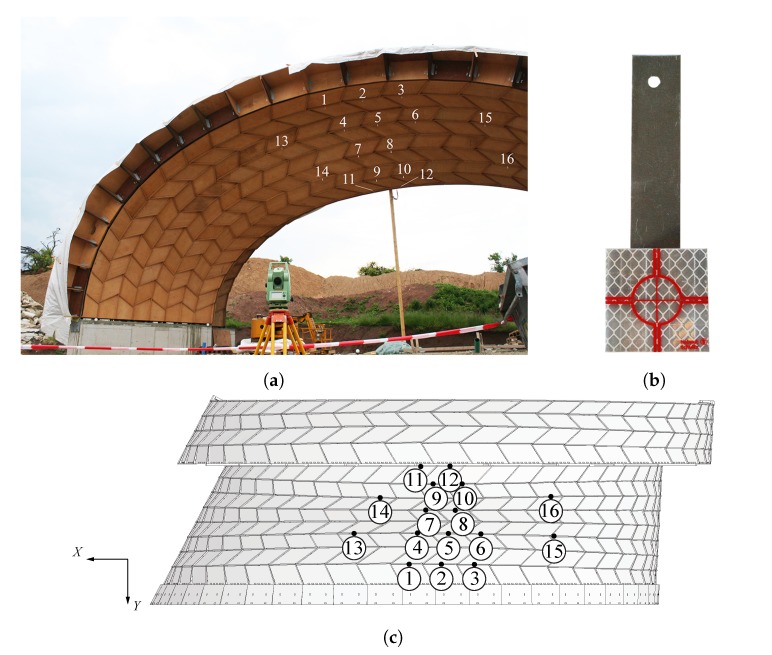
(**a**) A clear line of sight between the targets and the total station is required; (**b**) Reflective targets 3 × 3 cm; (**c**) Position of the 16 targets on arch n° 22.

Total stations are easy to use and have the advantage to measure distances and angles with high accuracy. However, they have a low data sampling rate and need an unobstructed line of sight between the targets and the total station [20]. In this study, measurements were taken at 16 different positions on the structure using an electronic total station Leica Geosystems TCR 305 (Leica Camera AG, Wetzlar, Germany). It has an accuracy (standard deviation) of 5 mm + 2 ppm for reflective targets to a range of 1.5 to 80 m (according to ISO 17123-4) and of 5″ (1.5 mgon) for angle measurements (according to ISO 17123-3) [28]. The total station was placed at a distance of about 10 m to the furthest measurement points in the *Y*-direction, in a clear line of sight to the 16 targets, as illustrated in Figure 5a. Microprismatic self-adhesive reflective targets of 3 × 3 cm and 0.5 mm thickness, both UV and weatherproof, were glued to aluminium plates (see Figure 5b), themselves screwed onto vertical panels of the structure at the 16 different positions indicated in Figure 5c.

To ensure that no movement of the device accidentally occurred before and after the measurements of the 16 targets, five reference targets were placed at stable locations. For both tests, successive measurements of the control points and the 16 targets were taken. It took about 10 min to acquire data for each sets of 16 points. All data was acquired onsite, taking about ten minutes per set of 16 points, and subsequently treated by computing the difference before targets position before and after loading for both tests. Sets of measurements for which successive control points measurements showed differences higher than 2 mm were discarded.

### 3.2. Terrestrial Laser Scanner

Terrestrial 3D laser scanners have been increasingly used for various applications. They allow fast acquisition of point clouds composed of millions of points through laser beam scanning. High resolution 3D images can therefore be obtained and complex environments can be reconstructed. In this study, a laser scanner FARO^®^ Focus${}^{S}$ 150 (FARO Technologies Inc., Lake Mary, FL, USA) was used to obtain point cloud measurements of the bottom surface of the arches. It scans at a measurement rate of 122,000 to 976,000 points per second with a visual field of 300°, vertically and 360°, horizontally, with an average 3D position accuracy of 3.5 mm at 25 m [29].

#### 3.2.1. Survey Procedure

**Figure 6:**
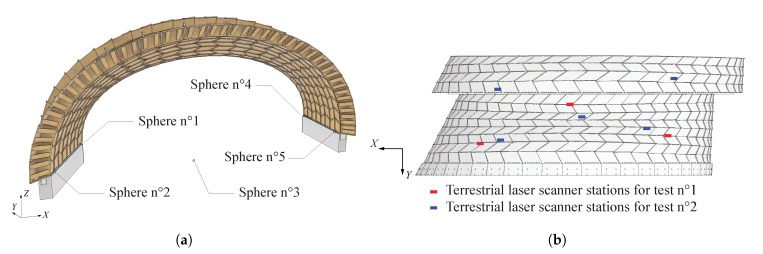
(**a**) Position of the five references spheres used for the registrations of the 3D laser scans; (**b**) Position of the stations of the 3D laser scans for test n° 1 (red) and test n° 2 (blue).

**Figure 7:**
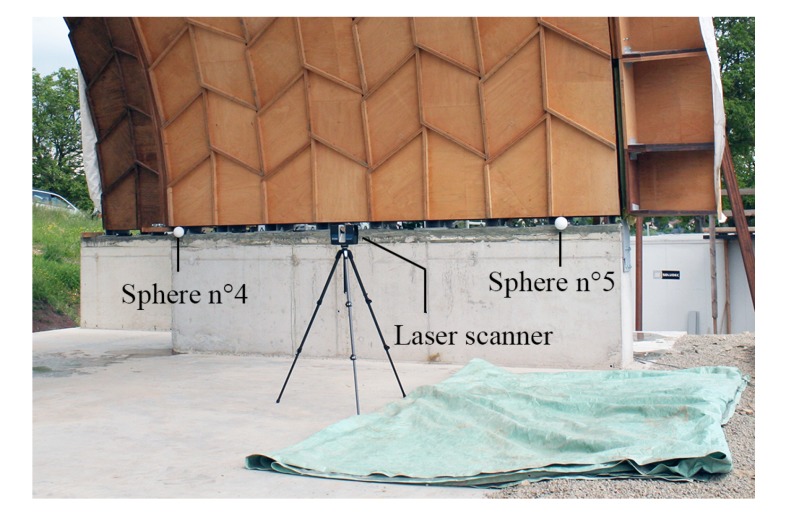
Spheres n° 4 and 5 and 3D laser scanner FARO^®^ Focus${}^{S}$ 150.

Before any measurement was taken, five artificial spherical targets were placed at the bottom corners and at the center of arch n° 22 (see Figure 6a). These survey markers were used for the registration of the scans. Their spherical shape prevents axis mapping mistakes [22]. Scans were acquired for both tests before and after loading. In order to collect data points over the entire bottom surface of the arches, they were performed from different stations, as indicated in Figure 6b. For test n° 1, for which arch n° 22 only was of interest, three scans were performed; for test n° 2, five scans were performed in order to obtain data points for both arch n° 22 and half arch n° 21. Spheres n° 4 and 5, as well as the laser scanner in one of the station position are illustrated in Figure 7.

Before each scan, the alignment level of the scanner was verified. Each scan with a 1/5 resolution took approximately 8 min. This resolution corresponds to an acquisition of 8192 points per 360° rotation, such that a full scan contains 28 million points (8192 × 3413) [29]. Photos were captured after each scan by the built-in digital camera of the laser scanner to obtain the exposure values of each point and hence the colors of the scan. Each point cloud contains millions of points with each point characterized by *x*, *y* and *z* coordinates and RGB values for their color.

#### 3.2.2. Registration and Pre-Processing

**Figure 8:**
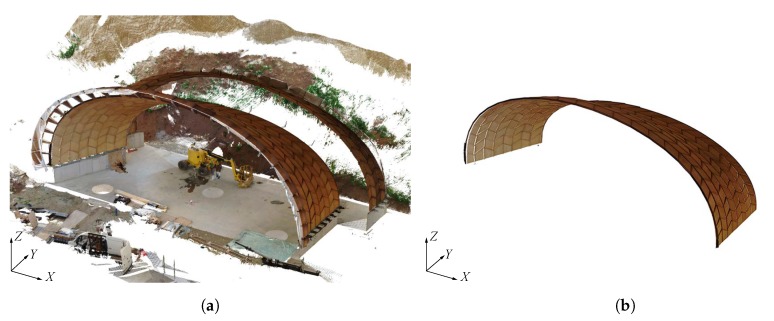
Pre-processing of the point clouds for Load case n° 1: (**a**) Raw point cloud; (**b**) Pre-processed point cloud reduce to 34 million points.

**Figure 9:**
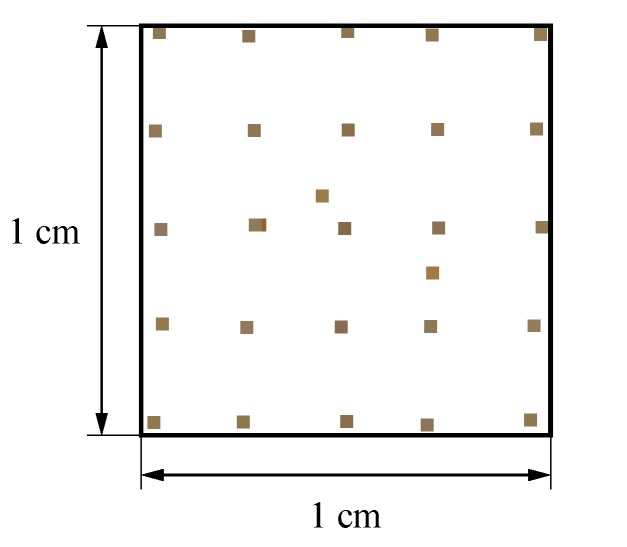
Area of 1 × 1 cm extracted from the pre-processed point cloud of Load case n° 1.

Compared to total station measurements, processing point clouds is not straightforward and can be cumbersome, as point clouds first need to be registered before computing distances [30]. In fact, since each point cloud station for the measurements has its own local coordinate system xyz, all points clouds have first to be represented in the same global coordinate system XYZ. Overall, the registration and point cloud pre-processing were completed in four steps. For scans belonging to the same load case, an automatic registration was first performed in the FARO^®^ SCENE software (FARO Technologies Inc., Lake Mary, FL, USA) using the spherical survey markers as references. For both tests, sets of scans before and after loading were then registered by superposing spheres, in this case spheres n° 2 and 5, for each load case by applying translations and rotations within FARO^®^ SCENE. Point clouds were manually pre-processed in a CAD software, in this case Rhinoceros^®^, version 6.0 (Robert McNeel & Associates, Seattle, WA, USA), to remove unnecessary regions around the scanned object of interest, as illustrated in Figure 8 for scan data of Load case n° 1. The resulting point cloud presented in Figure 8b contains about 34 million points and is illustrated for an area of 1 × 1 cm in Figure 9. According to Lee et al. [22], mapping errors of the sphere reference points need to be below 1 cm to ensure sufficient total accuracy of the measurements, in the few millimetres range for the present study. Registration were thus refined manually using the open source software CloudCompare [31] by aligning four point pairs manually picked from the reference cloud and the cloud to be compared.

In order to compare the point clouds measured without loading with the 3D model geometry of the structure used for fabrication, bottom surfaces of the model were retrieved. A triangular mesh, which can be processed with CloudCompare, was applied on these surfaces and a four point pairs alignment was then performed in CloudCompare.

#### 3.2.3. Distances Computation

**Figure 10:**
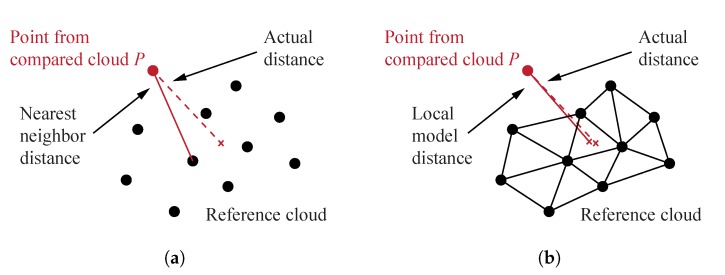
Cloud-to-cloud distances computation using: (**a**) the nearest neighbour distance; (**b**) methods in which a local model is applied between the nearest point and a definite number of neighbouring points.

Cloud-to-cloud distances were first computed using the nearest neighbour distance method, which consists in computing the Euclidean distance between a point of the compared cloud and the closest point of the reference cloud (see Figure 10a) [32]. Other methods consist in applying a mathematical model on the nearest point calculated with the Euclidian distance and a defined number of its neighbouring points in order to locally model a surface of the reference cloud. The distance between the point of the compared cloud and this mathematical model is then computed (see Figure 10b). Three mathematical models exist in CloudCompare: 2D1/2 Delaunay triangulation, least square best fitting plane and quadratic height function. Since the nearest neighbour method can be inaccurate if the reference point cloud is insufficiently dense, comparisons with the three distances computation methods available in CloudCompare were performed. For the comparison with the 3D model geometry, cloud-to-mesh distances computation was performed in CloudCompare by computing the distance between a point of the cloud and the nearest triangle of the mesh.

## 4. Numerical Model

### 4.1. Semi-Rigid Spring Model

**Figure 11:**
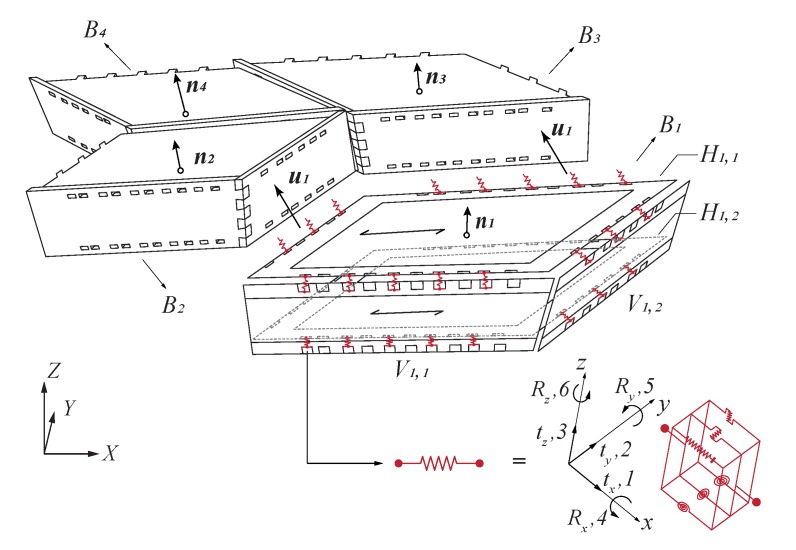
Semi-rigid spring model: plates were modelled as conventional shell elements and wood-wood connections as springs with six degrees of freedom (three translations and three rotations). Adapted from [26], Copyright (2019), with permission from Elsevier, based on the construction system developed by Robeller et al. [25].

A finite element model recently developed for the structural analysis of freeform timber plate structures using wood-wood connections was used in the present study [26]. In this model, plates were modelled as conventional shell elements and wood-wood connections were represented by springs with six degrees of freedom (three translations and three rotations) to take into account their semi-rigid behaviour (see Figure 11). A mesh composed of finite strain S4R elements was applied, using a fine mesh element size of 20 mm at the vicinity of the joints and a coarse mesh of 50 mm away from them, according to the mesh convergence study performed for small-scale prototypes [26]. As a manual generation of the model would have been too cumbersome, time-consuming and probably containing human errors, the model was generated automatically in the finite element analysis software Abaqus${}^{\mathrm{TM}}$, version 6.12 (Dassault Systèmes, Vélizy-Villacoublay, France) using the Abaqus Scripting Interface [33]. Linear kinematics were considered exclusively because of the low load levels applied on the structure. Geometrical nonlinearity was not considered as it was found to be essential only to precisely predict strains as well as displacements at higher loads, which was not the case in the present study [5].

The material was considered as a single orthotropic layer with material properties defined by three directions: the main direction corresponds to the longitudinal direction of the fibres, parallel to the grain; the second and third directions are perpendicular to the grain, along the veneer layer and across the thickness respectively. The model was built using the same mean orthotropic material properties for BauBuche-Q panels as for the investigations conducted on small-scale prototypes for density $\rho_{\mathrm{mean}}$, Young and shear modulus, *E* and *G* respectively, given by the manufacturer, as well as Poisson’s ratios ν retrieved from the literature (see Table 1) [26,27].

Regarding the semi-rigid properties of the connections, two sets of spring stiffness values were considered in this study. Axial, in-plane shear and out-of-plane shear stiffness, $k_{1}$, $k_{2}$ and $k_{3}$ respectively, as well as rotational stiffness about *y*, $k_{5}$, were retrieved from experimental tests for both sets [26,34,35]. Rigid rotational stiffness around *x* and *z*, $k_{4}$ and $k_{6}$ respectively, led to underestimated displacements when compared to experimental tests performed on small-scale prototypes. Therefore, simulations were performed with both $k_{4}$ and $k_{6}$ either rigid, by assigning them a high stiffness value of 10${}^{15}$ N·mm/°, or hinged, by assigning them a low stiffness value of 0.1 N·mm/°. In this manner, lower and upper bounds were obtained for displacements using a semi-rigid modelling of the connections. Spring stiffness values for all six degrees of freedom of the connections are summarized in Table 2.

### 4.2. Loads and Boundary Conditions

**Figure 12:**
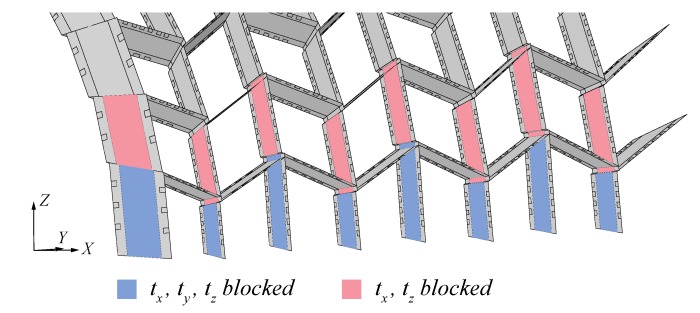
Numerical modelling of the boundary conditions applied on the coarse mesh area of vertical plates. Translations in *X*, *Y* and *Z* were blocked on the first row of boxes (in blue) and translations in *Y* and *Z* were blocked on the second row of boxes (in red).

At each end of the structure, boundary conditions were applied on the coarse mesh area of vertical plates, as illustrated in Figure 12. Translations in *X*, *Y* and *Z* were blocked on the first row of boxes and translations in *Y* and *Z* were blocked on the second row of boxes. Steel struts between the arches were modelled as beam elements connected to the arches using tie constraints between the struts beams and points on the structure, coupled to the edge of the plates. Gravity was applied in a first step and the cement bags were modelled in a second step by applying pressure loads corresponding to the exact number of bags on each box. Although tests occur after the action of gravity, its implementation allowed to take into account propagated effects of gravity. Weather conditions were characterized by wind speeds under 11 km/h during test n° 1 and under 17 km/h during test n° 2. Since these speeds correspond to light and gentle breeze on the Beaufort scale respectively, effects of wind were discarded in the model [36].

## 5. Results and Discussion

Displacements predicted with the numerical model were compared to the measurements taken at discrete target positions using the total station and to the displacement fields obtained at the bottom surface of the structure with the terrestrial laser scanner. To be able to establish a valid comparison, initial displacements due to gravity were subtracted from the final displacements obtained since experimental tests and measurements were executed after the disposition of the specimen on the supports. Furthermore, the theoretical geometry of the structure corresponding to the 3D design model was compared to the built prototype.

### 5.1. Discrete Target Positions

#### 5.1.1. Total Station Measurements

Three measurements of the 16 target positions were taken for each of the four load cases. Average differences of 0.05 mm ± 0.48% were observed between repetitive measurements and average values were therefore considered for each load case. Final displacements measured for the two tests are presented in Table 3. It can be observed that with the presence of half arch n° 21 connected through steel struts, displacements were increased by 15.1% ± 6.2% for targets 9 to 12 but were reduced for all other targets by 22.4% ± 7.3% in average. This could be explained by the fact that targets 9 to 12 were located close to the struts and therefore, the weight of half arch n° 21 increased the displacements locally. However, on the overall arch n° 22, displacements were reduced as the presence of half arch n° 21 rigidifies the structure.

#### 5.1.2. Validation of the Numerical Model

**Figure 13:**
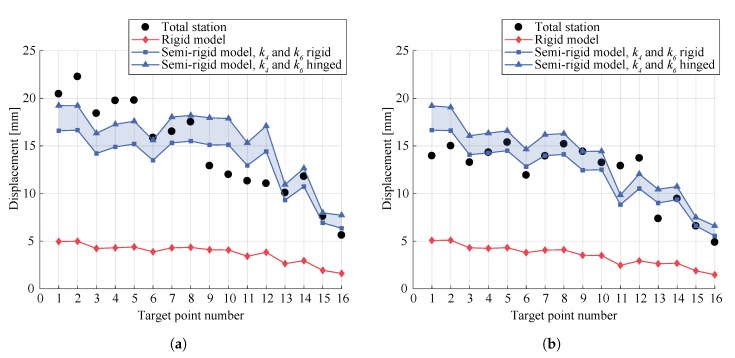
Comparison between the displacements measured with the total station and results predicted by a rigid model and semi-rigid models with *k*_4_ and *k*_6_ either rigid or hinged for: (**a**) test n° 1; (**b**) test n° 2. Target point numbers correspond to the 16 target points indicated in Figure 5c.

Figure 13 presents the comparison between the displacements measured with the total station and results predicted by the numerical model with rigid connections and semi-rigid models with $k_{4}$ and $k_{6}$ either rigid or hinged. First, it can be observed that a rigid model led to highly underestimated displacements with values 72.3% lower in average over all target points for both tests. Displacements obtained with the semi-rigid models were found to be of the same order of magnitude as the ones measured for both tests, with differences of 14.8% in average. However, for test n° 1, displacements of targets 9 to 12 were overestimated by 33.2% in average and underestimated by 15.5% for targets 1 to 6. These variations correspond to differences of 3.9 mm and 3.1 mm respectively and could be explained by the presence of caps, which was not considered in the numerical simulations, leading to an additional load that increases displacements in the area close to them and reduces displacements away from them. For test n° 2, highest variations between the model and the measurements also appeared for targets located in the vicinity of the caps and close to steel struts and could be explained by inaccuracies in the modeling of the connections between the steel struts and the structure.

### 5.2. Displacement Fields

#### 5.2.1. Cloud-Cloud Distances Computation

Registration and distances computation are important steps in the processing of the points clouds. Point clouds registration using the five spherical targets led to mapping errors of about 1 cm for different load cases. These errors were considered too high to provide a sufficient accuracy and, as suggested by Lee et al. [22], a manual registration was thus performed in the software CloudCompare considering four points at the bottom corners of arch n° 22.

**Figure 14:**
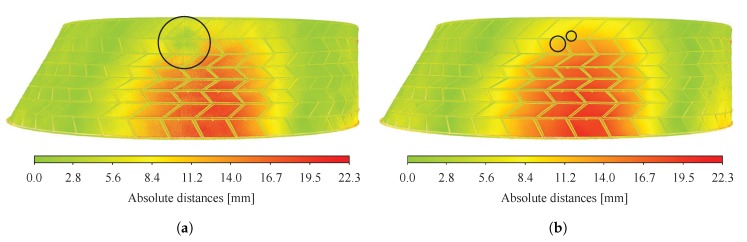
Distances computation for test n° 1 using the least square best fitting plane on: (**a**) the nearest point and its 6 neighbouring points; (**b**) the nearest point and its 99 neighbouring points.

For test n° 1, distances computed by applying local mathematical models on the nearest point and a defined number of its neighbouring points, as illustrated in Figure 10b, were compared with the ones obtained using the nearest neighbour distance (NND) method presented in Figure 10a. The three mathematical models available in CloudCompare were investigated, namely (i) 2D1/2 Delaunay triangulation, (ii) least square best fitting plane and (iii) quadratic height function. The displacement field obtained using (i) by fitting the model on the nearest point and its 6 neighbouring points was found to give similar results to the one obtained using the NND method. However, results using (ii) and (iii) on the nearest point and its 6 neighbouring points both led to noisy displacement fields with a large inaccurate area, as shown in Figure 14a for (ii). Analyses performed using (ii) and (iii) on the nearest point and its 99 neighbouring points increased the computing time and still led to small areas with errors, as illustrated in Figure 14b for (ii). Points clouds were thus found to be sufficiently dense to compute distances using the NND method and all other methods were therefore discarded for both tests.

#### 5.2.2. Total Station Measurements vs. Point Clouds

**Figure 15:**
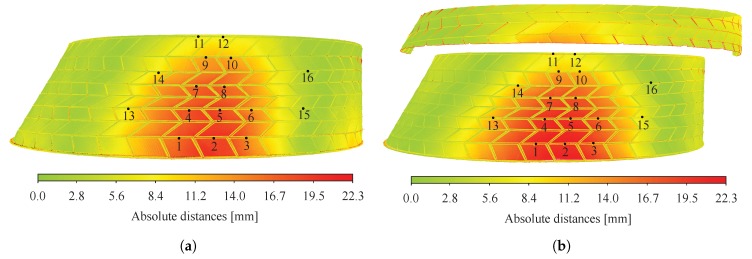
Distances computed from laser scans for: (**a**) test n° 1; (**b**) test n° 2. Numbered points indicate the 16 target points where displacements were measured using a total station.

Absolute distances computed with CloudCompare for test n° 1 and 2 are presented in Figure 15. Displacement fields obtained by laser scanning were first compared to total station measurements. It can be observed that point cloud distances computed were comparable for both tests, whereas higher differences could be observed with and without the presence of half arch n° 1 using a total station. Although precise values at discrete points are difficult to obtain with point clouds, it was found that overall values using the two measuring methods differed: at the 16 target positions, results from laser scans were found to be 2.9 mm and 0.7 mm lower than total station measurements on average for test n° 1 and 2 respectively. Furthermore, distances computed for specific target points gave differences of more than 60% between the two methods. For example, for test n° 1, points n° 15 and 16, corresponding to values of approximately 3 and 1 mm respectively for the point cloud, had measured displacements of 7.6 and 5.6 mm with the total station. Similarly, values computed at the target points 11 and 12 using the point clouds were found to be 37% and 4% in average lower than the total station measurements at these locations for test n° 1 and 2 respectively. Variations can be attributed to the registration of the point clouds to which results were shown to be very sensitive.

#### 5.2.3. Validation of the Numerical Model

**Figure 16:**
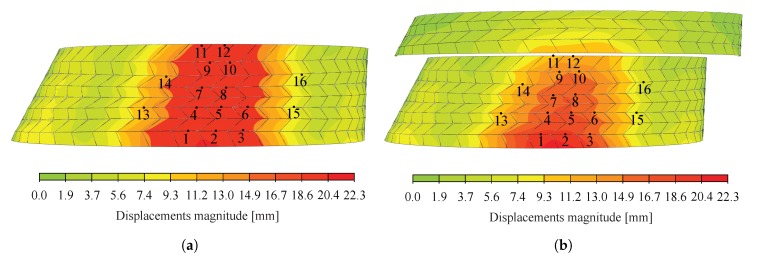
Displacements predicted by the semi-rigid numerical model, with rotational stiffness *k*_4_ and *k*_6_ hinged for: (**a**) test n° 1; (**b**) test n° 2. Numbered points indicate the 16 target points where displacements were measured using a total station.

Displacement fields at the bottom layer of the structure predicted by the semi-rigid model with rotational stiffness of the connections $k_{4}$ and $k_{6}$ hinged are presented in Figure 16a,b for test n° 1 and 2 respectively. Although uncertainties arose from the laser scans registration process, it can be observed that the same range of values was predicted by the semi-rigid model. However, the displacement field predicted was found to be closer to the one measured for test n° 2 (see Figure 15b and Figure 16b) than for test n° 1 (see Figure 15a and Figure 16a). For the latter, differences can be related to the presence of caps during the experimental tests (as explained in Section 5.1) and to point cloud registration errors.

#### 5.2.4. Evaluation of the Built Geometry

**Figure 17:**
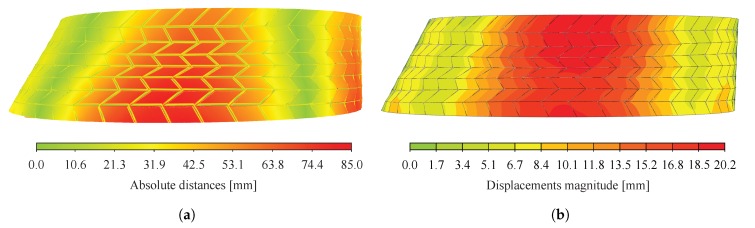
(**a**) Displacements between initial point cloud before loading (load case 1) and bottom surfaces extracted from the 3D design geometry; (**b**) Displacements obtained with the semi-rigid model with *k*_4_ and *k*_6_ hinged under selfweight only.

Additionally, the reconstructed structure of arch n° 22, obtained with laser scanning, was compared to the 3D design geometry of the CAD model to evaluate the gap between theoretical and as-built geometries. Absolute displacements between the point cloud of load case n° 1 and the bottom surfaces extracted from the 3D design geometry were first computed. Results of the cloud-to-mesh distance computation are presented in Figure 17a. Since the laser scans of the structure were executed for arch n° 22 under its own weight, displacements were computed for the selfweight load case to establish a valid comparison using the semi-rigid model with $k_{4}$ and $k_{6}$ hinged, leading to the largest displacements (see Figure 17b).

Large differences can be observed between the displacements predicted by the numerical model under selfweight only (see Figure 17b) and the cloud-to-mesh distance computation obtained for arch n° 22 unloaded (see Figure 17b), with maximum differences between the two of about 65 mm. Considering the results obtained in Section 5.1 and Section 5.2.3, these variations cannot be attributed only to the presence of caps in the tested prototypes, inaccuracies of the model and uncertainties linked to the point cloud registration, especially for the large displacements observed on the right side of the structure in Figure 17a. It was inferred that part of these large differences can be attributed to initial imperfections of the structure, such as geometrical imperfections, linked to the variance in structural dimensions of all the elements, and assembly tolerances. Geometric variability can be due to unavoidable machining errors, alterations from handling and transportation of the elements as well as moisture content variations. Although machining tolerances are expected to be low because of the precision of the 5-axis CNC cutting machine in the sub-millimetre range, tolerances can add up and lead to large geometric differences [37]. Initial imperfections might therefore need to be taken into account design calculations, as they can have a high influence on the structural stability of large-span structures [38]. As suggested by Liew et al. [39], adjusting the boundary conditions according to the as-built geometry could reduce these variations, in particular regarding the large displacements appearing on the right side of the structure.

## 6. Conclusions

In this paper, static loading tests were performed on large-scale freeform timber plate shells by applying a surcharge load of 1.5 kN/m${}^{2}$ on a defined area at the top of one structure. A comparison was made between displacements predicted by finite element models and displacements measured at 16 target positions with a total station and displacements fields obtained by computing distances between point clouds from laser scanning.

Total station measurements were found to give results with a higher precision of about 1–3 mm than laser scans for individual target points. However, point clouds from laser scans allowed to visualize displacement fields at the bottom surface of the structure such that the two methods are complementary. Regarding laser scans processing, a precise registration was found to have a significance importance as it can highly influence the results. Georeferencing laser scan data could help reducing errors linked to this registration step, as well as increasing the number of survey markers. Regarding the distances computation of the point clouds, using the nearest neighbouring distance was found to be sufficiently efficient compared to other methods using local mathematical models.

It was proven to be crucial to take into account the semi-rigidity of the connections to accurately predict the behaviour of the structure. In fact, a rigid modelling of the connections underestimated the displacements by about 72% ± 4% in average, whereas semi-rigid models gave results with a difference of about 15% ± 12% in average with respect to the total station displacements measured at the 16 target positions.

Cloud-to-mesh distances computed between the point cloud of arch n° 22 showed large displacements occurring without the application of surcharge loads. If part of these displacements can be attributed to inaccuracies of the numerical model and point cloud registration errors, the accumulation of fabrication and construction tolerances appears to be the main reason of these large differences observed. To tackle this issue, a precise assessment of all the parameters influencing the variations between 3D design model and as-built geometry is necessary.

In conclusion, the semi-rigid model developed was validated against full-scale experimental tests for the prediction of the displacements of the global structure in the linear elastic part, therefore allowing its serviceability limit state design. Furthermore, a semi-rigid model with hinged rotational components about *x* and *z* should be considered as it provides more conservative results.

## Figures and Tables

**Table 1:** Elastic properties used for orthotropic material model of 40 mm thick BauBuche Q panels. Reprinted from [26], Copyright (2019), with permission from Elsevier.

Property	Symbol	Value	Unit
Elastic modulus 11	$E_{11}$	13,200	N/mm${}^{2}$
Elastic modulus 22	$E_{22}$	2200	N/mm${}^{2}$
Elastic modulus 33	$E_{33}$	2200	N/mm${}^{2}$
Shear modulus 12	$G_{12}$	820	N/mm${}^{2}$
Shear modulus 13	$G_{13}$	430	N/mm ${}^{2}$
Shear modulus 23	$G_{23}$	430	N/mm${}^{2}$
Poisson’s ratio 12	$\nu_{12}$	0.365	–
Poisson’s ratio 13	$\nu_{13}$	0.464	–
Poisson’s ratio 23	$\nu_{23}$	0.726	–
Density	$\rho_{\mathrm{mean}}$	800	kg/m${}^{3}$

**Table 2:** Spring stiffness values used in the finite element model for each of the 6 degrees of freedom. Adapted from [26], Copyright (2019), with permission from Elsevier.

Symbol	Component	Value	Units
$k_{1}$	translation *x*	416.81	N/mm
$k_{2}$	translation *y*	15,009.24	N/mm
$k_{3}$	translation *z*	9489.04	N/mm
$k_{4}$	rotation around *x*	10${}^{15}$/0.1	N·mm/°
$k_{5}$	rotation around *y*	170,190.41	N·mm/°
$k_{6}$	rotation around *z*	10${}^{15}$/0.1	N·mm/°

**Table 3:** Displacements of the 16 targets measured with the total station for test n° 1 and 2. Averaged values on three repetitive measurements for each load case.

Displacements [mm]			
Target n°	Test n° 1	Test n° 2	Difference [%]
1	20.5	14.0	−31.8
2	22.3	15.0	−32.6
3	18.4	13.3	−27.9
4	19.8	14.3	−27.5
5	19.8	15.4	−22.3
6	15.9	11.9	−24.8
7	16.5	13.9	−15.6
8	17.5	15.2	−13.2
9	12.9	14.4	+11.7
10	12.0	13.2	+10.4
11	11.3	12.9	+14.1
12	11.1	13.7	+24.1
13	10.1	7.4	−27.0
14	11.8	9.5	−19.8
15	7.6	6.6	−13.4
16	5.6	4.9	−13.2

**Figure A1:**
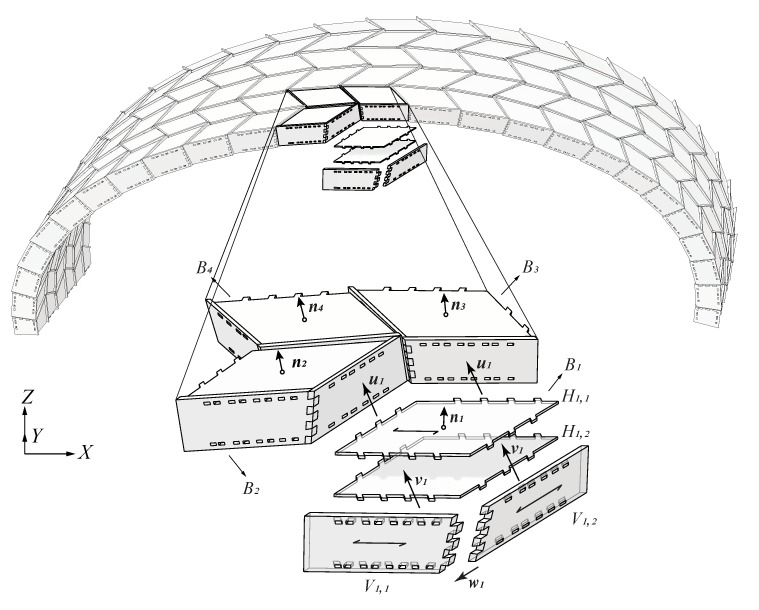
Assembly system of double-layered double-curved timber plate shells developed by Robeller et al. [25]

## Appendix A. Assembly System

The assembly system used for double-layered double-curved timber plate shells, which have been recently developed by Robeller et al. [25] and applied to the Annen Plus SA head office project, is illustrated in Figure A1. All plates of the structure are assembled using multiple tab-and-slot joints (MTSJ) as well as through-tenon multiple tab-and-slot joints (MTSJ-TT) [3,40].

The two layers of each shell are composed of thin timber plates, namely $H_{i,1}$ and $H_{i,2}$ for top and bottom layers respectively, characterized by a dihedral angle close to 180° between neighbouring plates. Vertical plates $V_{i,1}$ and $V_{i,2}$ are introduced to connect these horizontal plates through MTSJ-TT, with tenons integrated in horizontal plates and slots in vertical plates. Vertical plates are assembled with dovetail MTSJ. Each arch can also be seen as an assembly of four-sided hexahedra-shaped boxes $B_{i}$ composed of two horizontal plates, corresponding to the two layers of the structure, and two vertical plates allowing their assembly through MTSJ-TT. Each boxes has thus two remaining open sides that are closed by neighbouring boxes, such that vertical plates are shared between them.

All MTSJ used in the structure are single-degree-of-freedom joints associated with unique insertion vectors. Vertical plates $V_{i,1}$ and $V_{i,2}$ are first assembled following the insertion vector $w_{i}$ and horizontal plates $H_{i,1}$ and $H_{i,2}$ are subsequently inserted in the vertical plates following $v_{i}$. Four-sided boxes thus formed individually are then subsequently assembled together along $u_{i}$. The interlocking of the plates allows the remaining degree-of-freedom of the connections to be blocked. Using this assembly system, arches are formed following a unique assembly path.
